# Fluorescent Lectins for Local *in Vivo* Visualization of Peripheral Nerves

**DOI:** 10.3390/molecules19079876

**Published:** 2014-07-08

**Authors:** Gijs Hendrik KleinJan, Tessa Buckle, Danny Michel van Willigen, Matthias Nathanaël van Oosterom, Silvia Johara Spa, Harmen Egbert Kloosterboer, Fijs Willem Bernhard van Leeuwen

**Affiliations:** 1 Interventional Molecular Imaging Laboratory, Department of Radiology, Leiden University Medical Hospital, Albinusdreef 2, PO Box 9600, 2300 RC Leiden, The Netherlands; 2 Department of Head and Neck Oncology, The Netherlands Cancer Institute-Antoni van Leeuwenhoek Hospital, Plesmanlaan 121, 1066CX, Amsterdam, The Netherlands; 3 Department of Bionanotechnology, Wageningen University, PO Box 8038, 6700EK Wageningen, The Netherlands

**Keywords:** imaging agents, fluorescence, peripheral nerves, lectins, surgical guidance

## Abstract

Damage to peripheral nerves caused during a surgical intervention often results in function loss. Fluorescence imaging has the potential to improve intraoperative identification and preservation of these structures. However, only very few nerve targeting agents are available. This study describes the *in vivo* nerve staining capabilities of locally administered fluorescent lectin-analogues. To this end WGA, PNA, PHA-L and LEL were functionalized with Cy5 (λ_ex max_ 640 nm; λ_em max_ 680 nm). Transfer of these imaging agents along the sciatic nerve was evaluated in Thy1-YFP mice (n = 12) after intramuscular injection. Migration from the injection site was assessed *in vivo* using a laboratory fluorescence scanner and *ex vivo* via fluorescence confocal microscopy. All four lectins showed retrograde movement and staining of the epineurium with a signal-to-muscle ratio of around two. On average, the longest transfer distance was obtained with WGA-Cy5 (0.95 cm). Since WGA also gave minimal uptake in the lymphatic system, this lectin type revealed the highest potential as a migration imaging agent to visualize nerves.

## 1. Introduction

Damage to the peripheral nervous system (PNS) is a surprisingly common complication after surgery (e.g., prostatectomy, colorectal surgery and the removal of head and neck tumors) [1,2,3,4,5]. Many peripheral nerves are encountered within the surgical field and their intraoperative identification is often difficult. Unfortunately, trauma to these nerves can lead to chronic function loss and, as such, can negatively influence the quality of life of patients [6,7].

In recent years intraoperative fluorescence imaging was introduced to increase contrast between a target lesion and the surrounding anatomy. Fluorescent dyes such as fluorescein and indocyanine green (ICG) are routinely used in the evaluation of perfusion and optical detection of cancerous lesions [8]. During sentinel lymph node biopsies the addition of fluorescence, incorporated in a hybrid imaging agent, was shown to improve optical detection after local administration of a tracer [9,10]. 

In the preclinical setting fluorescence has been used to image (peripheral) nerves. Unfortunately, fluorescent dyes alone have not yet shown the required specificity for nervous tissue necessary for their application in the clinic [11]. To increase specificity, a targeting moiety can be introduced that selectively binds to cells expressing a specific biomarker. This approach has already been applied frequently in cancer imaging [12,13]. Compounds that stain the entire nervous system have been described in literature, e.g., FAM-NP 41 [14]. Unfortunately, intravenous administration may also increase the chance of systemic toxicity. Since the latter is a great concern, we reasoned that, in analogy to the clinically applied sentinel node procedure [9,10], local tracer administration could provide an alternative means to highlight nerves while reducing the (systemic) dose [15]. Following a local administration, only the nerves in the anatomy that is being surgically interrogated will be stained.

Lectins are a group of proteins, with size varying between 4–10 nm, which have shown promise as imaging agents [16,17,18,19]. Previously, these proteins have been used for (trans synaptic) axonal tracing in *ex vivo* histological research to identify neuronal pathways; labeling occurred with horse radish peroxidase (HRP) [16,18,20,21]. Lectins are able to bind to specific sugar groups (oligosaccharides), leading to affinity for proteoglycans (PGs) present on the extracellular matrix of peripheral nervous tissue [22]. These PGs consist of a protein core to which one or more glycan chains are attached [23], creating five distinct PGs; chondroitin sulfate proteoglycan (CSPG), heparan sulfate proteoglycan (HSPG), keratan sulfate proteoglycan (KSPG), dermatan sulfate proteoglycan (DSPG) and hyoluronan proteoglycan (HP) [24,25]. For examples of different lectins and their corresponding accessory sugar moieties and PGs, see Table 1.

Reasoning that a local administration of lectins may also provide *in vivo* migration along peripheral nerves, in this study Cy5-labeled lectin derivatives were evaluated for their value *in vivo*. Performance of the lectins was scored by: (i) measuring the transfer distance of the lectins along the course of the nerve; (ii) determining the fluorescence signal measured in the nerve to the signal in the surrounding tissue (signal-to-background-ratio; SBR) and (iii) evaluating the binding mode after *ex vivo* incubation of nerve tissue.

**Table 1:** Lectins and their corresponding binding sugar moiety/proteoglycan [22,26,27,28].

Lectin	Sugar Moiety	PG	Molecular Weight (KD)
Triticum Vulgaris (Wheat germ agglutinin; WGA);	β-d-GlcNAc, Neu5Ac	HSPG, KSPG, HP	36
Arachis Hypogaea (Peanut lectin; PNA)	Gal β (1–3)GalNAc	CSPG, DSPG	110
Phaseolus Vulgaris Leucoagglutinin(Red kidney bean; PHA-L)	Antennary branched β (1–6) GlcNAc	HSPG, KSPG, HP	120
Lycopersicon Esculentum (Tomato lectin; LEL)	[GlcNAc β (1–4)]_2−4_	HSPG, KSPG, HP	71

## 2. Results and Discussion

### 2.1. Labeling Efficacy

**Figure 1:**
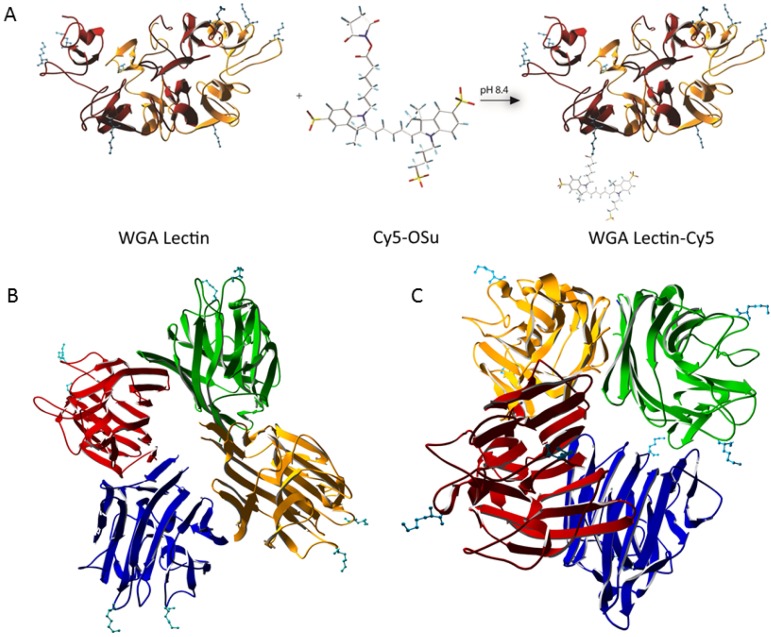
Crystal structures and method of labeling. (**A**) WGA, consisting of two subunits, with four readily available lysines per subunit, covalently attached to sulphonated Cy5-OSu. (**B**) PHA-L, consisting of four subunits, with two readily available lysines per subunit. (**C**) PNA, consisting of four subunits forming a tetrahedral structure, with two readily available lysines per subunit. To the best of our knowledge, the crystal structure for LEL (tomato lectin) is unknown.

**Figure 2:**
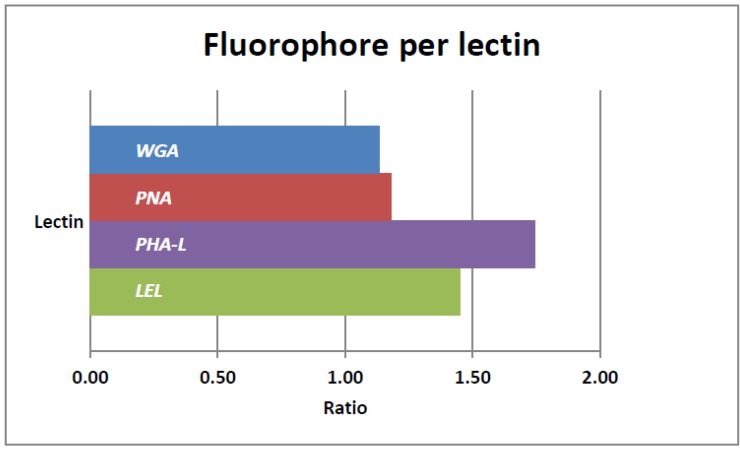
Cy5/Lectin labeling ratio per lectin.

To study the value of lectins for *in vivo* visualization of nerves following a local tracer administration, the four different lectins used in this study (Table 1) had to be labeled with a fluorescent dye. We chose the far-red dye Cy5 based on its previous use in preclinical and clinical studies on fluorescence guided surgery [13,29]. To ensure optimal comparison between the lectins, all four were labeled via an identical labeling protocol (Figure 1A). The reaction between Cy5-OSu and the free lysine groups available on the lectins (Figure 1B,C lysine groups in blue), resulted in a successful fluorescent labeling with corresponding labeling ratios (Figure 2). Via absorption spectroscopy the average Cy5/lectin ratio was determined to be 1.38 ± 0.24, meaning that , at least one fluorophore was attached to the protein scaffold. Slight differences in labeling efficiency (Figure 2) can presumably be explained by the amount of available lysines on the lectins and their spatial conformation within the protein structure. As depicted in Figure 1, WGA has four available lysines per subunit, namely LYS33, LYS88, LYS134 and LYS149, whereas PHA-L and PNA have only 2, respectively LYS129/LYS215 and LYS77/LYS112.

By increasing the ratio of fluorophores per lectins, the fluorescence signal in the stained nerve, and the resulting SBR, could potentially increase. The fluorescence signal will, however, not only be limited by the number of available binding sites on the molecule, but also by the quenching effect that will occur when these dyes are being placed within 10 nm of each other [30]. The maximum distances between available lysines were measured using the crystal structures (Figure 2) and Swissprot software. Here we found that, in the case of WGA, no more than one fluorophore per lectin is desired, as the maximum lysine-to-lysine distance is approximately 7.3 nm. For PNA and PHA-L, the maximum distance between the lysines is approximately 8.4 and 9.7 nm. As such, an average of 1.5 fluorophores per lectin is considered the optimal labeling ratio.

### 2.2. In Vivo Migration

**Figure 3:**
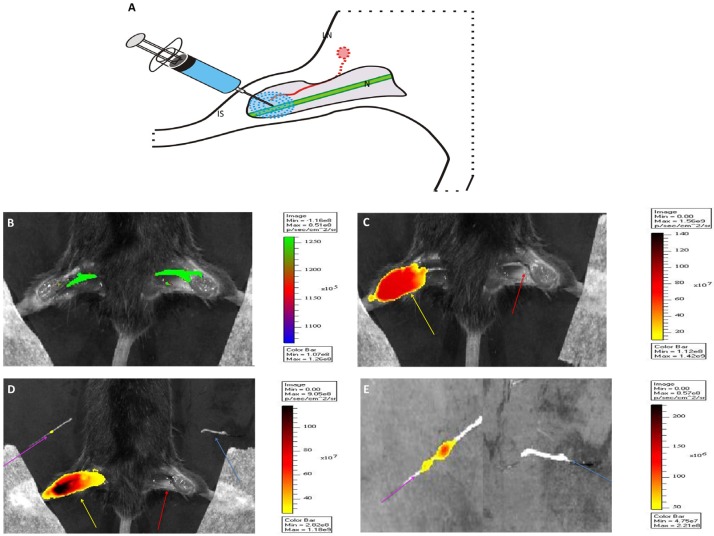
*In vivo* distribution of locally injected lectins (**A**) Schematic representation of the local injection of Cy5-lectins and the corresponding migration path. The injection site (IS) and sciatic nerve (N) are depicted in blue and green respectively. The lymphatic tract leading from the IS to the lymph node (LN) is shown in red. (**B**) Illustration of the YFP signal in the nerves (green) which served as control for the localization of the nerves. Representative images of the injection site before (**C**) and after (**D**) removal of the nerve, and excised nerves (**E**). In each of these pictures, yellow arrows -the injection site; purple arrows -fluorescence signal from injection site and nerve, red arrows -the control side nerve and light blue arrows -the control nerve.

Good visualization of a nerve following local administration of a fluorescently labeled lectin requires migration of the imaging agent along the course of the nerve. Staining of the nerve will commence at the site of injection and continue along the length of the migration (Figure 3A). This process was studied *in vivo* using transgenic THY-1 YFP mice in which the nerves themselves are fluorescent in the 520–550 nm region, thereby providing an internal reference for the migration path (Figure 3B; nerve in green). An intramuscular injection was aimed at the distal part of the sciatic nerve in the hind leg (Figure 3A). From this location, the lectin-analogues were allowed to migrate for 24 h. After this period, consecutive imaging at Cy5 settings (ex 633 nm, em 650–700 nm) was performed before and after dissection of the muscle tissue that surrounds the sciatic nerve (Figure 3C,D).

As the images in Figure 3 demonstrate, the background signal emitted by the injection site was so intense that initial removal of this tissue was required to enable detection of fluorescence in the nerve (Figure 3E). No fluorescence was observed in the control nerve in the opposite leg (Figure 3, red arrow). Clinically the drawback of an intense signal at the injection site is also encountered during, e.g., sentinel lymph node biopsy, a procedure that relies on the local injection of a radiocolloid and/or fluorescent dye that subsequently migrates through the lymphatic system [31]. In this application, the signal from the injection site sometimes overshines lymph nodes located in close proximity [32]. Here, technical solutions such as changes in the imaging equipment and imaging software have been successfully applied to circumvent this problem [33,34].

**Figure 4:**
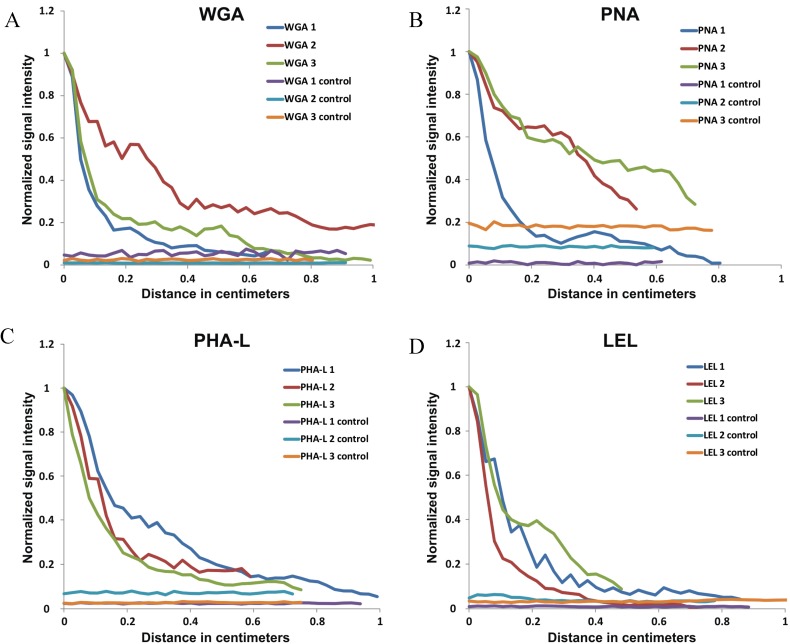
Migration curves. Normalized curves of (**A**) WGA, (**B**) PNA, (**C**) PHA-L and (**D**) LEL show the migration of the lectins along the individual nerves. On the y-axis the normalized intensity signal is depicted, on the x-axis the migration distance (cm).

Analysis in the different animals revealed that all four lectins migrated along the nerve, but slight differences in the efficiency could still be recorded (Table 2, Figure 4). To do this, the maximal retrograde transfer length determined from the normalized curves produced by MATLAB, was set at the point where the (average) signal in the nerve was equal to the signal in the unstained control.

**Table 2:** Migration distance and signal to background ratios.

	WGA	PNA	PHA-L	LEL
Average migration distance (cm) (SD)	0.95 (0.20)	0.72 (0.13	0.81 (0.20)	0.72 (0.20)
SBR nerve_control_ (Average + SD)	2.08 (1.11)	1.72 (0.32)	1.86 (0.70)	4.88 (2.48)
SBR nerve_muscle_ (Average + SD)	1.86 (1.00)	1.42 (0.12)	1.12 (0.13)	1.26 (0.46)
SBR_Ipsilateral lymph node_ (Average + SD)	0.61 (0.26)	0.70 (0.06)	3.73 (1.36)	1.11 (0.87)
SBR_Contralateral lymph node_ (Average + SD)	0.45 (0.42)	0.55 (0.17)	0.79 (0.33)	0.86 (0.62)

SD = Standard deviation; cm = centimeters; SBR = signal to background ratio.

The value of the lectins as nerve visualizing imaging agents will increase with an increasing migration distance. For all four lectins-analogues, the normalized curves show a fast decrease in signal intensity along the nerve (Figure 4). Although not significant (*p*-value = 0.659), WGA gave the best migration properties (0.95 cm; Figure 4A) compared to PHA-L (0.81 cm; Figure 4C), and PNA and LEL (both 0.72 cm; Figure 4B,D). Local identification of peripheral nerves may help surgeons to navigate around the complex (nerve) anatomy in the head-and-neck area; numerous (small) nerves are located within a surgical field of 2 × 2 cm [35]. One may question the value of a “mere 1 cm migration” along the nerve within this field of view. However, during our clinical studies in the field of fluorescence guided sentinel node biopsy we already found that fluorescence may provide surgical guidance towards sentinel nodes located within 1 cm of the injection site [36]. In our view this suggests that the compound described here may already provide value. Chemical modification of these imaging agents (e.g., functionalization, solubility) may increase the migration along the nerves which will further broaden the application of this approach.

### 2.3. Signal Intensity

*In vivo* visualization efficacy depends on the intensity of the fluorescence signal emitted by the nerve-bound lectins and the difference between the signal in the nerve and the surrounding tissue. 

The degree of visibility of the individual nerves was determined by calculating the SBR in the lectin-stained nerve compared to the control nerve (SBR_control_; Table 2). The SBR of Cy5 labeled WGA and LEL was shown to be 1.1 to 2.8-fold higher than the ratio found for PNA and PHA-L. The SBR between the signal in the nerve and the signal in the control muscle tissue (SBR_muscle_; Table 2) reveals how well a stained nerve can be potentially detected when surrounded by non-stained tissue. With WGA a ratio of 1.86 was obtained while with LEL and PHA-L SBR values of 1.26 and 1.12 were found respectively (Table 2). Based on the assumption that a SBR of approximately 2 is desirable for efficient *in vivo* visualization [37], it can be concluded that WGA would be the most effective imaging agent in this setting. 

Alternative Drainage Routes

After local injection, edema is created at the site of injection. As a result, part of the injected volume may be transported through the lymphatic system to the lymph nodes (Figure 3A). Since in this experiment the aim was to achieve maximal selective staining of the nerves, such distribution via the lymphatics was unwanted. To determine the degree of lymphatic clearance through the lymphatic system, the SBR between the fluorescence signal in the nerve and inguinal lymph nodes (LN) was calculated (signal nerve/signal LN; Table 2). The SBR between the fluorescence signal in the ipsilateral lymph node and the injection site was shown to be higher for LEL and PHA-L, compared to WGA and PNA. Similar results were obtained when comparing the fluorescence signal in the injection site and the contralateral inguinal lymph node. Although the lymphatic drainage makes lectins like PHA-L (SBR = 3.73) candidates for sentinel node imaging, the lower degree of lymphatic clearance of WGA and PNA makes these two compounds more suitable for nerve specific staining. 

### 2.4. Evaluation of Binding Mode

**Figure 5:**
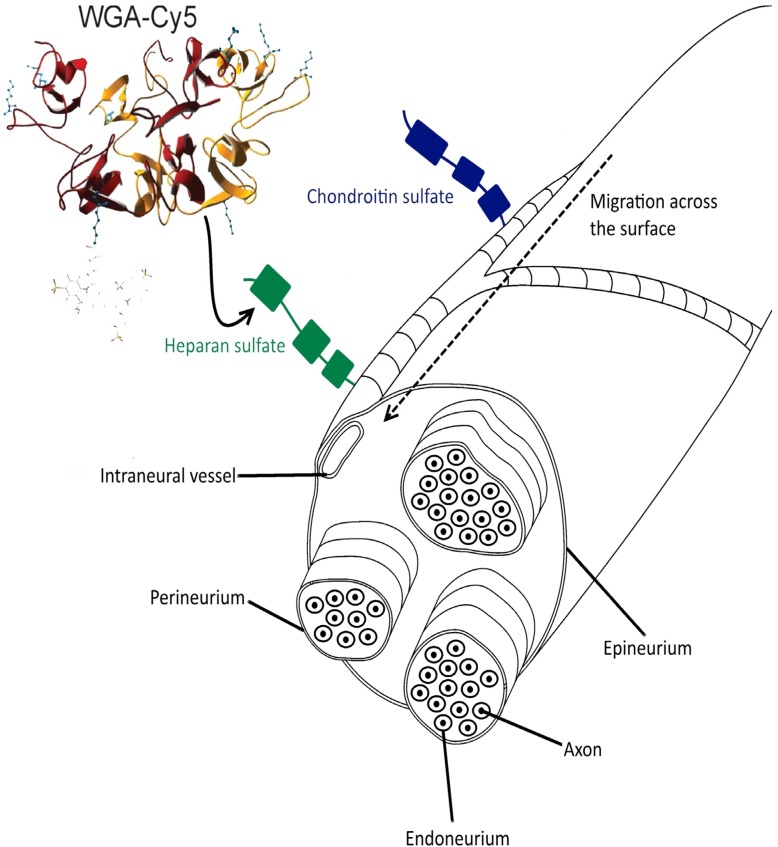
Schematic overview peripheral nerve wherein Cy5/ WGA lectin binds to PG’s located on the epineurium and extracellular matrix.

Staining of a nerve by fluorescent labeled lectins is influenced by the availability and accessibility of PGs (See Table 1). Peripheral nerves, such as the sciatic nerve, are surrounded by a dense layer of connective tissue. This layer, the epineurium, encloses multiple nerve fascicles as well as fatty tissue and blood vessels. Smaller branches of these blood vessels penetrate into the perineurium, a protective sheath serving as blood-nerve barrier that surrounds the different fascicles. Within these fascicles, nerve fibers are bundled together. Each fiber is again surrounded by its own protective layer (the endoneurium; a thin layer of connective tissue) that encloses individual axons (Figure 5) [4,38].

To assess the binding mode of the individual lectins after local *in vivo* injection, the nerves were analyzed *ex vivo* using fluorescence confocal microscopy. This experiment demonstrated that in all cases, after intramuscular injection, a Cy5 signal could be detected at the location of the epineurium throughout the course of the nerve. The epineurium, which consists out of collagen type 1 (connective tissue), expresses the sugar moieties targeted by the different lectins (Table 1) [39,40].

**Figure 6:**
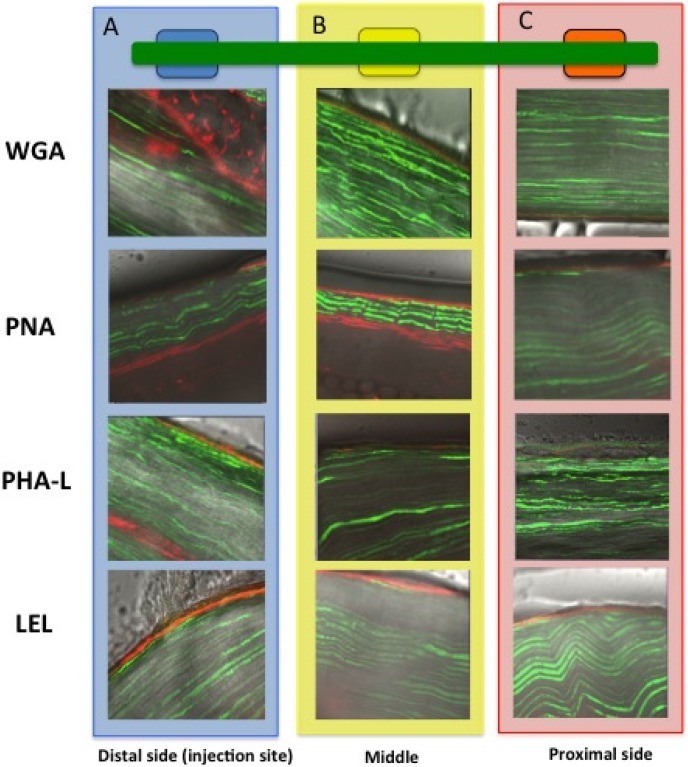
Binding mode after *in vivo* local administration. The fluorescence signal in nerves from Thy-1 YFP mice (YFP in green and Cy5 in red) was traced from (**A**) the injection site to (**B**) the middle and (**C**) the proximal side of the nerve. In all cases, staining of the epineurium was observed with a decrease in signal when the distance from the injection site increased.

For all lectins, the intensity of the staining was the highest at the distal side of the nerve (also representing the injection site, Figure 6A). While fluorescence could still be detected in the middle part (Figure 6B) and the proximal part of the nerve (Figure 6C), the intensity of the signal decreased at longer migration distances. The latter is in agreement with the findings shown in Figure 4, which illustrates a gradual decrease in signal along the course of the nerve.

**Figure 7:**
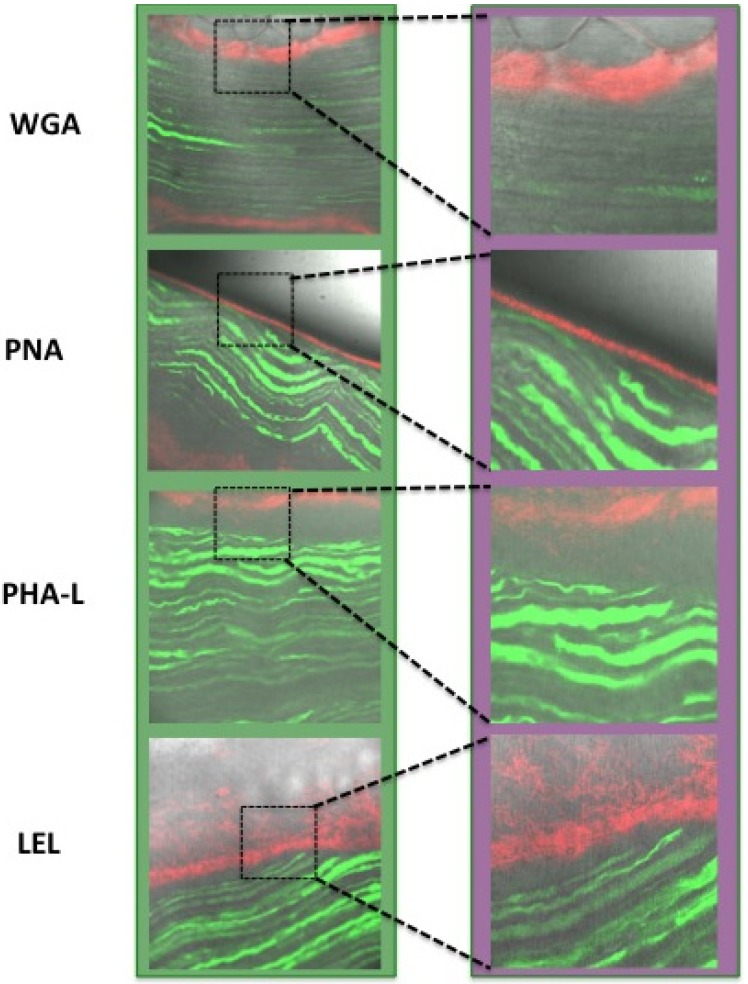
*Ex vivo* incubation. *Ex vivo* incubation confirmed the *in vivo* localization of staining. (YFP in green and Cy5 in red)

To determine if the migration process influences the binding of the lectin-analogues, nerves were also stained *ex vivo*. To this end, excised nerves were placed in a solution containing (one of) the Cy5- labeled lectins. By doing so, exposure to the imaging agent may not be limited to the epineurium. This, however, resulted in a highly similar staining pattern compared to which is observed after *in vivo* incubation (Figure 7); Staining of the epineurium and not of other structures within the nerve (e.g., the axons) was observed. As staining of the epineurium will not affect the signal conduction within the nerve and the neurons itself, this feature can be considered favorable for *in vivo* use. This may also provide an advantage over neuronal tracing using neurotoxins [15].

Previous studies have shown that CSPG and HSPG are present on the endoneurium, epineurium and perineurium (Figure 5) of the peripheral nerves [41,42]. WGA the best binding lectin in this study was shown to have the highest affinity for HSPG, KSPG and HP [23,24,25,43]. However, the lectins PHA-L and LEL share its affinity for HSPG (see Table 1). For that reason, something other than the affinity for HSPG seems to drive the difference in migration. Most likely, the size of the lectin is a determining factor during the migration, where smaller molecules show increased migration speed. As shown in Table 1 and Figure 1, WGA is the smallest of the lectins evaluated; it only consists out of two subunits, while PHA-L and PNA consist out of four subunits (structure of LEL is unknown, see above).

## 3. Experimental Section

### 3.1. Experimental Equipment

The fluorescent dye Cy5 was purified with an HPLC 1525 Pump and 2489 UV/Vis detector (Waters, Etten-Leur, The Netherlands), using a Reprosil-Pur 120 C18-AQ 10 µm column (Dr.Maisch GmbH, Ammerbuch-Entringen, Germany) using a 60 min gradient of H2O/MeCN (0.1% TFA) 95:5 – 5:95. Reaction mixtures were filtered using 10K Amicon Ultra-0.5 mL Centrifugal Filters (Merck Millipore, Billerica, MA, USA). Centrifugation was performed with an Eppendorf 5415D centrifuge (Eppendorf Nederland B.V., Nijmegen, The Netherlands). Absorption was measured with a Nanodrop ND-1000 spectrophotometer (Thermo Scientific, Wilmington, DE, USA). Protein figures were made using SwissProt PDB viewer and Adobe Illustrator CS6 (Adobe Systems Inc., San Jose, CA, USA). The WGA, PNA, PHA-L lectins were purchased from Sigma Aldrich (St. Louis, MO, USA). The LEL lectin was purchased from Vector Laboratories Inc. (Burlingame, CA, USA).

An IVIS Spectrum animal fluorescence scanner (Perkin Elmer, Waltham, MA, USA) was used for evaluation of *in vivo* migration. The acquired IVIS data was analyzed with Living Imaging Acquisition and Analysis software. Furthermore, for analysis of migration and intensity signals in the nerves, the raw IVIS data was analyzed with MATLAB software (Mathworks Inc., Torrance, CA, USA). An SP5 Confocal Microscope (Leica Microscopes B.V., Rijswijk, The Netherlands) was used for fluorescence confocal microscopy. Nerve tissue was placed on glass bottom dishes (Ø 35 mm dish, No. 1.5, Ø 14 mm glass surface, MatTek, Ashland, MA, USA) prior to imaging. Confocal images were acquired with Leica LAS AF software. Statistical analysis was performed with SPSS Statistics data analysis (Version 20, SPSS Inc., Chicago, IL, USA). 

### 3.2. Synthesis of Sulphonated Cy5-OSu

Sulphonated Cy5 was synthesized according to previously described methods [44]. The OSu activation was performed in DMSO (800 μL), using HSPyU (5 eq) and DIPEA (10 eq). After completion of the reaction, water (0.1% TFA) was added (3.2 mL) and the mixture was purified by RP-HPLC. The collected fractions were lyophilized and a dark blue solid (Sulphonated Cy5-OSu) was obtained (28.1 mg, 46%). MS MALDI-TOF Calculated: *m/z* 860.0, found: *m/z* 862.8. Of this dark blue solid a stock solution was prepared (0.97 mM in DMSO).

### 3.3. General Conjugation Procedure

WGA (Figure 1A), PHA-L (Figure 1B) and PNA (Figure 1C), and LEL were each dissolved in 200 µL of 0.1 M phosphate buffer pH 8.4 and Cy5-OSu stock solution was added. The aim was to achieve a labeling ratio of 1–1.5 fluorophores per lectin. The necessary equivalents of dye per lectin were calculated using Equation (1) and the labeling ratios that were determined by a test-conjugation (Table 3). The test-conjugation was performed to determine how many equivalents of fluorophore were necessary to achieve the optimal labeling ratio. As a starting point, 5 equivalents of fluorophore were used and the ratios were calculated according to Equations (2)‒(4). The obtained ratios were inserted in formula 1 and the appropriate amounts of equivalents calculated. Note: the compounds obtained during the test-conjugations were not used for further experiments. The reaction was repeated with the calculated equivalents of fluorophore (Table 4) and the reaction mixture was gently stirred at room temperature for 2 h. Hereafter, the mixture was transferred to a 10KD cut-off filter (Amicon) and centrifuged at 14.000 G. Saline (400 µL) was added and the mixture was again centrifuged, this was repeated until the filtrate was colorless. Subsequently, the residue (blue liquid) was collected.

Equation (1): Calculation of needed equivalents of fluorophore

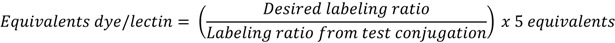



**Table 3:** Amounts used for final conjugation.

	WGA	PNA	PHA-L	LEL
Labeling ratio from test-conjugation	1.08	1.25	0.75	1.48
Amount of lectin (nmol)	27.8	9.1	4.2	14.1
Amount of fluorophore (nmol)	191.8	54.6	35.3	70.5
Equivalents of dye/lectin	6.9	6.0	9.9	5.0

Equation (2): Fluorophore concentration

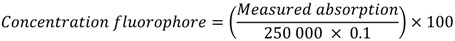



Equation (3): Amount of fluorophore

*Total amount of fluorophore = Concentration fluorophore × Total sample volume*

Equation (4): Labeling ratio

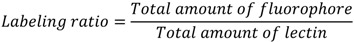



**Table 4:** Calculation data.

	WGA	PNA	PHA-L	LEL
Measured absorption	0.068	0.019	0.008	0.028
Fluorophore concentration (nmol/μL)	0.272	0.076	0.032	0.112
Total sample volume (µL)	139	139	128	150
Total amount of fluorophore (nmoL)	37.81	10.56	4.10	16.80
Total amount of lectin (nmoL)	27.80	9.10	4.15	14.10

### 3.4. Labeling Ratio Analysis

The samples were diluted 100× and 2 µL of each sample was used for the absorption spectroscopy measurement (Nanodrop). The labeling ratio was calculated according to the following equations, using and/or generating the data in Table 3.

### 3.5. *In Vivo* Distribution

To study the *in vivo* distribution of the lectins, Thy1-YFP mice were used (n = 12). In these genetically modified mice, neurons are fluorescently labeled with YFP. The YFP signal can be used as an internal control regarding the location of (peripheral) nerves. Per animal, 20 μL (32 μM lectin-Cy5) was injected in the thigh muscle and the injection was aimed for the sciatic nerve (Figure 1A). All animals tolerated the lectin injections without evidence for systemic toxicity, this is in concordance with previous literature where lectins were injected in the fore limb [17]. Animal experiments were conducted according to Dutch law and after approval was obtained from the institutional animal ethics committee.

Twenty four hours after injection, the animal was sacrificed, whereafter fluorescence imaging with IVIS was performed to visualize the distribution of the lectins throughout the sciatic nerve. The contralateral side was taken as a negative control. Fluorescence images were acquired with Cy5 filter settings (λ ex max, 640 nm; λ em max, 680 nm), while the YFP signal was measured at λ ex max, 465 nm and λ em max, 520 nm. To depict the staining sites and locations, the nerves in mice were imaged three times with the IVIS, first with intact skin, second with the skin removed and last with the muscle structures surrounding the nerve removed. Both the injection site nerve and the control nerve were collected from the mice for *ex vivo* imaging. The fluorescence signal in the inguinal lymph nodes was analyzed to study the migration of the lectins through the lymphatic system (Figure 3A). Both the ipsilateral and the contralateral inguinal lymph nodes were removed for analysis.

The measured intensities (photons/s/cm^2^/sr) were quantified by Living Imaging Acquisition and Analysis software. The total flux (photons/s/cm^2^/sr) in the nerves was measured by drawing a region of interest around both nerves. The signal to background ratios were measured by: a) dividing the signal of the injection site nerve by the control nerve and b) dividing the signal of the injection site nerve by the signal in the surrounding muscle tissue on the contralateral side.

MATLAB software was used to generate a signal profile along the sciatic nerve (injection site and control), based on the raw IVIS data. A virtual pixel-wide line was drawn along the *ex vivo* sciatic nerve to measure the signal intensity. The counts measured and generated by the software were converted into a normalized curve for all the experiments. The maximum of these curves was determined as the site of injection and the normalization was done by setting the peak corresponding to the Cy5 fluorescent signal along the injection nerve, at y = 1. From punctum maximum (y = 1), the length of the curve over the x-axis was measured in pixels, and thereafter converted into centimeters. A signal (curve) higher than the control nerve signal was designated as fluorescent signal in the nerve (Figure 4). With this technique, the migration of the x-axis in number of pixels (0.0268 cm per pixel) was calculated. The average migration distance of all lectins were compared using a Kruskal Wallis test, a *p*-value of < 0.05 is accepted as statistical significance.

### 3.6. *Ex Vivo* Incubation and Fluorescence Confocal Microscopy

The location of the signal was analyzed using fluorescence confocal imaging. Nerves were assessed after local injection and the control nerves were used for *ex vivo* incubation (1 h) experiments. For analysis with confocal microscopy, the nerves were washed with PBS after the incubation and placed on glass bottom dishes, which were mounted on the confocal microscope. YFP was excited with a 488 nm laser and emission was detected between 520 nm and 550 nm. Cy5 was excited with a 633 nm laser and emission was detected between 650 nm and 700 nm. The location of the lectin was assessed with the Cy5 signal in/on the nerve.

## 4. Conclusions

Fluorescent lectins were shown to be potential candidates for *in vivo* visualization of the peripheral nerves. Using local administration, WGA was shown to have the best properties of the four different lectins tested. Since all four lectins only showed staining in the nerve epineurium, the chance of inducing systemic toxic side effects after administration of these agents will be limited. 
